# Morbidity and Mortality Among People Living With HTLV‐1: A 30‐Year Retrospective Analysis in a Brazilian Cohort

**DOI:** 10.1002/jmv.70849

**Published:** 2026-02-20

**Authors:** Elizabeth Souza Neves, Otavio Melo Espíndola, Raquel de Vasconcelos Carvalhaes de Oliveira, Ana Claudia Celestino Bezerra Leite, Marco Antonio Sales Dantas Lima, Marcus Tulius Teixeira Silva, Rafael Osellame, Pedro Abrahão Pinheiro Guimarães, Ricardo Vezzani Batista, André Luiz Anjos Oliveira, Abelardo Queiroz Campos Araújo

**Affiliations:** ^1^ Instituto Nacional de Infectologia Evandro Chagas Fundação Oswaldo Cruz Rio de Janeiro Brazil

**Keywords:** HTLV‐1, HTLV‐1‐associated myelopathy/tropical spastic paraparesis, morbidity, mortality

## Abstract

HTLV‐1 infection is a chronic condition associated with the development of malignancies, inflammatory, and neurological disorders. In this study, the causes of hospitalization and mortality were assessed in a cohort of 508 individuals living with HTLV‐1 followed over a 30‐year period. Overall mortality was 8.5% in this population and increased to 15% among patients with HTLV‐1‐associated myelopathy/tropical spastic paraparesis (HAM/TSP), a major neurological syndrome characterized by motor, sensory, and urinary dysfunction. Hospitalization was required by 147 participants of the cohort (28.9%), and this increased to 40.2% (121 of 301) among those with HAM/TSP. Patients were admitted at least once, with a mean of two admissions per patient. Hospitalization was associated with HAM/TSP, particularly the degree of motor disability and HIV coinfection. Infectious diseases were the leading cause of both hospitalization (66.7%) and death (69%), most commonly urinary tract infections, pneumonia, and infected pressure ulcers. In addition, mortality was also directly related to the intensity of motor impairment and the number of hospitalizations. HAM/TSP was associated with an increased risk of hospitalization and readmission. Additionally, greater motor impairment was linked to higher risks of both hospitalization and mortality, while elevated HTLV‐1 PVL was found to contribute to an increased risk of death. In conclusion, these findings highlight the importance of prevention and timely management of secondary infections to reduce morbidity and mortality in individuals living with HTLV‐1, particularly those affected by HAM/TSP, which frequently present urinary dysfunction and pressure ulcers.

## 1 Introduction

The human T‐cell lymphotropic virus type 1 (HTLV‐1), described in 1980 as the first retrovirus associated with human diseases [1], is linked to a broad clinical spectrum. The most common HTLV‐1‐associated conditions are adult T‐cell leukemia/lymphoma (ATLL), HTLV‐1‐associated myelopathy/tropical spastic paraparesis (HAM/TSP), and uveitis [2]. Other neurological conditions, including dysautonomia, cognitive impairment, and polyneuropathy [3], in addition to non‐neurological manifestations such as infective dermatitis, arthritis, and polymyositis, have also been reported [4, 5].

Several aspects of HTLV‐1 infection pathogenesis remain unclear, including the determinants of clinical presentations and the fact that fewer than 5% of infected individuals develop disease [2]. The clinical course of ATLL is typically rapid and associated with high mortality, whereas HAM/TSP is a chronic, progressive, and disabling condition, but is not generally considered a direct cause of death. Instead, secondary complications such as recurrent urinary tract infections (UTIs) due to neurogenic bladder, pneumonia, and infection of pressure ulcers contribute substantially to morbidity, particularly in patients with motor impairment related to spinal cord injury [6, 7]. However, the impact of these complications on morbidity and mortality in people living with HTLV‐1, independently of ATLL, has not been systematically assessed.

In this study, we aimed to describe the causes and factors associated with hospitalization and death among HTLV‐1‐infected patients without ATLL, providing evidence and insights for the development of preventive strategies, to help reduce the costs of recurrent hospitalizations, and ultimately improve the quality of life of individuals living with this retroviral infection.

## 2 Materials and Methods

### 2.1 Study Design and Population

A longitudinal study was conducted in a historical open cohort that followed 508 individuals living with HTLV‐1 at the Instituto Nacional de Infectologia Evandro Chagas, Fundação Oswaldo Cruz, Rio de Janeiro, Brazil, between January 1, 1992, and December 31, 2022. This study protocol was approved by the Institutional Research Board (protocol number 71046117.6.0000.5262).

Patients were eligible if they met the following criteria: (i) definitive diagnosis of HTLV‐1 infection, with positive serology by ELISA and confirmation by Western blot (WB) or PCR; (ii) regular follow‐up, defined as no discontinuation of more than 4 months for symptomatic patients and more than 1 year for HTLV‐1 asymptomatic carriers (HAC); and (iii) absence of ATLL.

### 2.2 Exposure Variables and Clinical Assessment

Exposure variables included sex, age, overall clinical classification as HAC, HAM/TSP, other neurological abnormalities (ONA), and non‐neurological manifestations (NNM); degree of neurological disability, HIV co‐infection, and peripheral blood HTLV‐1 proviral load (PVL). Neurological disability was assessed using the Expanded Disability Status Scale (EDSS) of Kurtzke [8], which ranges from 0 (normal neurological examination) to 9 (complete loss of mobility and communication). HAM/TSP was diagnosed by two independent neurologists in accordance with the World Health Organization criteria [9].

HTLV‐1 PVL was determined by quantitative PCR with TaqMan probes in a Cepheid Smart Cycler II real‐time PCR equipment, as previously described [10]. The PVL was calculated as the percentage of HTLV‐1‐infected cells in the population of peripheral blood leukocytes (PBL) using the following formula: [*tax* copies/(*β‐globin* copies/2)] × 100. The assay presented a lower limit of detection of one infected cell (one *tax* copy) per 10 000 PBL.

HIV diagnosis was performed using two immunoassays, followed by confirmation using WB or quantitative RT‐PCR; participants with indeterminate results had a new blood sample collected after 30 days to repeat the diagnosis algorithm to obtain a definitive result.

The HTLV‐1 PVL was dichotomized at the median value, age was stratified into groups of less than 60 years and 60 years or older, and EDSS scores were categorized according to mobility status: ≤ 5 (independent mobility), 6 (use of one or two walking aids), and ≥ 7 (restricted mobility, able to walk up to 5 m, essentially requiring a wheelchair).

### 2.3 Statistical Analysis

Descriptive statistics included absolute and relative frequencies for categorical variables and summary measures, including mean and standard deviation, median, and interquartile range (IQR) for continuous variables. Associations between categorical variables (sex, HIV co‐infection, hospitalization, and death) were assessed using Fisher's exact test. Two primary outcomes were considered: death and hospitalization. The normal distribution of continuous variables was assessed using the Shapiro–Wilk test. Comparisons of continuous variables (age, EDSS, and HTLV‐1 PVL) by outcome were performed with the Mann–Whitney test, except for age, which was evaluated with *T*‐test. The variables with significant associations were included in the survival analysis, and the age variable was added as a control variable due to theoretical relevance. An exploratory survival analysis was conducted to assess both time to death and time to first hospitalization outcomes, utilizing the Kaplan‐Meier method. HTLV‐1 presentation curves were compared using the log‐rank test, and the 95th percentiles of time to survival were reported. Since overall survival exceeded 50%, median survival times could not be determined. Cox proportional hazards models were fitted in both single and multi‐covariate models to analyze each outcome: “time to death” and “time to first hospitalization”. After, we analyzed the time to recurrence of hospitalization (rehospitalization), considering an extended Cox model named Prentice, Williams, and Peterson (PWP) model [11]. The analyses considered the interval from the first quantification of HTLV‐1 PVL (baseline level) to either the three outcomes of interest (death, first hospitalization, or rehospitalization) or the censoring date (December 31, 2022, if the patient was alive). Age, HTLV‐1 clinical presentation as asymptomatic, HAM/TSP, and HTLV‐1 PVL were retained in multicovariate models based on theoretical relevance. Schoenfeld residuals did not reject the proportional assumption. Results were expressed as crude and adjusted hazard ratios (HRs) with 95% confidence intervals (CIs). A two‐sided *p*‐value < 0.05 was considered statistically significant. All analyses were conducted using *R* software v4.2.1 and the survival package for survival analysis (R package version 3.5‐8, https://CRAN.R-project.org/package=survival).

## 3 Results

A total of 508 patients were included, with a median age of 48.0 years (IQR 40.2–56.3), and the majority were female (*n* = 319, 62.8%). Clinical presentation comprised 162 asymptomatic carriers (31.9%), 307 with HAM/TSP (60.4%), 20 with ONA (3.9%), and 19 with NNM (3.7%). Median EDSS score was 4.0 (IQR 0.0–6.0). HIV co‐infection was identified in 39 patients (10.3%). According to data available for 473 of 508 patients, the median HTLV‐1 PVL at inclusion in the cohort was 5.2% of HTLV‐1‐infected cells/PBL (IQR 1.6%–9.4%).

During follow‐up, 103 patients (20.3%) died, of whom 84 had HAM/TSP, accounting for 81.6% of deaths. Hospitalization occurred in 147 patients (28.9%), with a median of two admissions (IQR 1.0–3.0; range, 1–11). Symptomatic patients, including those with HAM/TSP, ONA, and NNM, had more admissions (up to 11 hospitalizations) compared with asymptomatic carriers (up to 4 hospitalizations) (*p* < 0.001). Among symptomatic patients (*n* = 346), 340 had information on hospital admission, and 126 (37.1%) were hospitalized at least once, while 214 (62.9%) were never hospitalized. Of the 329 hospitalization episodes, most (*n* = 228, 69.3%) were related to secondary infections, predominantly UTIs (*n* = 131, 57.5%), pneumonia (*n* = 42, 18.4%), and pressure ulcers (*n* = 25, 11.0%).

The cause of death could be determined in 82 of 89 symptomatic cases. Infectious diseases were responsible for three‐quarters of deaths (*n* = 62, 75.6%), mainly respiratory (*n* = 25), urinary tract (*n* = 21), and pressure ulcers (*n* = 9) infections, sepsis of undetermined origin (*n* = 3), central nervous system infection (*n* = 1), other dermatosis (*n* = 1), and one case of atypical mycobacteriosis and peritoneal carcinomatosis. Non‐infectious causes accounted for 24.4% (*n* = 20) of deaths. Factors associated with hospitalization and death are summarized in Tables 1 and 2. Hospitalization was associated with HTLV‐1 clinical presentation, HTLV‐1 PVL, HIV coinfection, and EDSS (Table 1). Death was also associated with these factors, except for HIV coinfection, but also with age and the number of admissions (Table 2). No associations with sex were observed.

**Table 1:** Distribution of risk factors according to hospitalization in a cohort of individuals living with HTLV‐1 between 1992 and 2022, Rio de Janeiro, Brazil.

Characteristics	Categories	Hospitalized		Non‐hospitalized		*p*‐value
		*n*		*n*		
Sex	Male	53	36.1%	135	38.0%	0.753^b^
	Female	94	63.9%	220	62.0%	
Age (years)	Mean ± SD	147	49.0 ± 11.8	355	47.2 ± 12.7	0.142^c^
HTLV‐1 clinical presentation	HAC	21	14.3%	141	39.7%	**< 0.001** b
	HAM/TSP	121	82.3%	180	50.7%	
	ONA/NNM	5	3.4%	34	9.6%	
HTLV‐1 PVL^a^	Median (IQR)	124	7.2% (2.6–10.5%)	343	4.8% (1.1–8.8%)	**0.001** d
HIV coinfection	Yes	17	16.2%	22	8.3%	**0.040** b
	No	88	83.8%	244	91.7%	
EDSS score	Median (IQR)	147	6.0 (4.0–7.0)	355	2.0 (0.0–6.0)	**< 0.001** d

Abbreviations: EDSS, Expanded Disability Status Scale; HAC, HTLV‐1 asymptomatic carrier; HAM/TSP, HTLV‐1‐associated myelopathy/tropical spastic paraparesis; IQR, interquartile range; NNM, non‐neurological manifestations; ONA, other neurological abnormalities; PVL, proviral load; SD, standard deviation.

^a^ HTLV‐1 PVL represents the percentage of infected cells in the population of peripheral blood leukocytes.

^b^ Fisher's exact test.

^c^ Student *t*‐test.

^d^ Mann–Whitney test. Differences were considered significant when *p*‐value < 0.05 (in bold).

**Table 2:** Distribution of risk factors according to death in a cohort of individuals living with HTLV‐1 between 1992 and 2022, Rio de Janeiro, Brazil.

Characteristics	Categories	Death		No death		*p*‐value
		*n*		*n*		
Sex	Male	35	34.0%	154	38.0%	0.520^b^
	Female	68	66.0%	251	62.0%	
Age	Mean ± SD	103	52.8 ± 12.1	405	46.6 ± 12.3	**< 0.001** c
HTLV‐1 clinical presentation	HAC	14	13.6%	148	36.5%	**< 0.001** b
	HAM/TSP	84	81.6%	223	55.1%	
	ONA/NNM	5	4.9%	34	8.4%	
HTLV‐1 PVL^a^	Median (IQR)	82	7.5% (4.1–11.6%)	391	4.8% (1.2–8.9%)	**< 0.001** d
HIV coinfection	Yes	4	4.9%	35	11.8%	0.110^b^
	No	77	95.1%	261	88.2%	
EDSS score	Median (IQR)	103	6.0 (4.0–7.0)	405	3.0 (0.0–6.0)	**< 0.001** d
Hospitalizations per patient	Median (IQR)	103	2.0 (1.0–3.0)	405	0.0 (0.0–0.0)	**< 0.001** d

Abbreviations: EDSS, Expanded Disability Status Scale; HAC, HTLV‐1 asymptomatic carrier; HAM/TSP, HTLV‐1‐associated myelopathy/tropical spastic paraparesis; IQR, interquartile range; NNM, non‐neurological manifestations; ONA, other neurological abnormalities; PVL, proviral load; SD, standard deviation.

^a^ HTLV‐1 PVL represents the percentage of infected cells in the population of peripheral blood leukocytes.

^b^ Fisher's exact test.

^c^ *T*‐test.

^d^ Mann–Whitney test. Differences were considered significant when *p*‐value < 0.05 (in bold).

The time of follow‐up of participants in this open cohort varied up to 404 months. Given the prolonged clinical course of HTLV‐1 infection, overall survival exceeded 50%; therefore, the median time to event could not be estimated. Accordingly, the 95th percentile of time to the outcomes of interest (death and first hospitalization), defined as the time by which 95% of individuals in each group had not yet experienced the event, was used to enable meaningful comparisons between groups. A longer survival time was noted in the HAC group (95th percentile = 193 months), followed by individuals with manifestations other than HAM/TSP (ONA and NNM) (95th percentile = 86 months). The shortest survival periods were observed among those diagnosed with HAM/TSP (95th percentile = 54 months) (log‐rank *p*‐value < 0.001). The median time to first hospitalization could not also be determined due to prolonged survival; consequently, the 95th percentile was used, corresponding to 11 months. A shorter time to first hospitalization was observed for individuals with HAM/TSP (95th percentile = 5 months), followed by those with other manifestations (ONA and NNM) (95th percentile = 75 months), and HAC (95th percentile = 96 months) (log‐rank *p*‐value < 0.001).

In the multicovariate Cox model for first hospitalization (Table 3), absence of HIV co‐infection was protective (HR = 0.27). Patients with HAM/TSP had an increased risk compared with asymptomatic carriers (HR = 2.51), and those with EDSS ≥ 6 had a greater risk than those with EDSS ≤ 5 (HR = 1.95 and HR = 4.49, respectively). Age and HTLV‐1 PVL were not significant predictors for hospitalization. In the multicovariate Cox model for death (Table 3), neurological disabilities (HR = 2.04 for EDSS = 6 vs. ≤ 5, and HR = 3.19 for EDSS ≥ 7) and higher HTLV‐1 PVL (HR = 1.70, > 5.2% vs. < 5.2%) increased the risk of death. Age and HIV coinfection were not significant predictors of death. Regarding rehospitalization, the multicovariate analysis indicated that HAM/TSP was the sole factor associated with an increased risk of rehospitalization compared to HAC (HR = 1.82) (Table 3).

**Table 3:** Hazard effects estimated by the Cox model for time to first hospitalization and death.

Characteristics	Categories	Time to first hospitalization (*n* = 346)		Time to death (*n* = 471)		Time to rehospitalization (*n* = 285)	
		Crude HR (CI 95%)	Adjusted HR (CI 95%)	Crude HR (CI 95%)	Adjusted HR (CI 95%)	Crude HR (CI 95%)	Adjusted HR (CI 95%)
Age (years)	< 60	1.00	1.00	1.00	1.00	1.00	1.00
	≥ 60	1.21 (0.79–1.83)	0.58 (0.31–1.09)	**2.46 (1.59–3.80)**	1.36 (0.80–2.33)	**1.43 (1.08–1.89)**	1.41 (0.92–2.18)
HIV coinfection	Yes	1.00	1.00	—	—	1.00	1.00
	No	**0.54 (0.31–0.87)**	**0.27 (0.14–0.50)**	—	—	**1.79 (1.26–2.55)**	1.17 (0.65–2.10)
HTLV‐1 clinical presentation	HAC	1.00	1.00	1.00	1.00	1.00	1.00
	HAM/TSP	**4.22 (2.65–6.72)**	**2.51 (1.22–5.13)**	**4.57 (2.54–8.21)**	1.68 (0.77–3.66)	**2.31 (1.63–3.28)**	**1.82 (1.04–3.18)**
	ONA/NNM	1.04 (0.39–2.75)	1.20 (0.39–3.70)	**1.77 (0.63–4.96)**	1.47 (0.51–4.27)	1.52 (0.70–3.27)	1.80 (0.68–4.76)
EDSS score	≤ 5	1.00	1.00	—	—	1.00	1.00
	6	**2.47 (1.65–3.71)**	**1.95 (1.05–3.59)**	**2.77 (1.67–4.59)**	**2.01 (1.07–3.77)**	**1.32 (1.01–1.73)**	1.07 (0.69–1.66)
	≥ 7	**6.05 (4.11–8.91)**	**4.49 (2.46–8.19)**	**6.94(4.37–11.02)**	**3.19 (1.66–6.12)**	**2.11 (1.64–2.70)**	1.29 (0.82–2.03)
HTLV‐1 PVL^a^	≤ 5.2%	1.00	1.00	1.00	1.00	1.00	1.00
	> 5.2%	**1.52 (1.06–2.17)**	1.14 (0.73–1.77)	**2.45 (1.53–3.93)**	**1.70 (1.03–2.79)**	1.24 (0.99–1.57)	1.09 (0.79–1.51)

Abbreviations: CI 95%, 95% confidence interval; EDSS, Expanded Disability Status Scale; HAC, HTLV‐1 asymptomatic carrier; HAM/TSP, HTLV‐1‐associated myelopathy/tropical spastic paraparesis; HR, hazard ratio; NNM, non‐neurological manifestations; ONA, other neurological abnormalities; PVL, proviral load.

^a^ HTLV‐1 PVL represents the percentage of infected cells in the population of peripheral blood leukocytes; significant differences are indicated in bold. Cox proportional hazards models were fitted in both single (crude) and multicovariate (adjusted) forms for each outcome.

## 4 Discussion

HAM/TSP is often considered a condition of relatively low mortality, but it substantially impacts the quality of life of patients [12]. In addition to motor impairment, affected individuals may experience complications such as neurogenic bladder and pressure ulcers. A potential link between HTLV‐1 infection and higher mortality rates has been shown [13, 14, 15]; however, ATLL is the only condition currently recognized as a direct cause of death. Studies assessing morbidity and mortality in people living with HTLV‐1 often include participants who develop ATLL, which strongly influences hospitalization and death outcomes given its severity [16].

Approximately one‐third of patients with HAM/TSP experienced, on average, two hospitalization episodes. The rate of hospitalization in this group was higher than that observed in HAC or in individuals with other HTLV‐1‐related manifestations. Hospital readmissions occurred in 36.4% of patients, exceeding the 23% reported in a multicenter study conducted in Spain [17]. The primary causes of hospitalization were infections, particularly UTIs, respiratory infections, and those associated with pressure ulcers, which is consistent with previous reports [18, 19]. Indeed, most infection‐related hospitalizations were attributed to UTIs. The high prevalence of UTIs among patients with HAM/TSP is likely associated with neurogenic bladder dysfunction, including detrusor‐sphincter dyssynergia and increased post‐void residual volume. This association is further compounded by prolonged use of indwelling catheters in patients who are in advanced stages of the disease and wheelchair‐bound [20, 21]. Regarding respiratory infections, an increased incidence of community‐acquired pneumonia and pulmonary tuberculosis has been observed among individuals living with HTLV‐1 compared to uninfected controls [15, 19, 22]. Among Australian Aboriginal populations, bronchiectasis and chronic bronchitis/bronchiolitis were significantly more common in individuals with HTLV‐1 infection [23, 24]. Skin disorders, particularly xerosis and seborrheic dermatitis, have also been associated with HTLV‐1 infection [25]. In contrast, despite the extensive reports in cases of spinal cord injury, pressure ulcers are rarely reported in these patients [26, 27]. Multiple biological processes might influence the burden of secondary infections in patients with HAM/TSP. Emerging evidence has implicated urinary, gut, and cutaneous dysbiosis in increased colonization by opportunistic skin or uropathogens, thereby facilitating recurrent or invasive infections [28, 29]. In addition, chronic HTLV‐1 infection is associated with persistent immune activation and immune dysregulation, resulting in a strong shift towards Th1 response and immunodominance to HTLV‐1 antigens, which can promote low‐grade systemic inflammation and impair effective pathogen clearance [30].

Bivariate analysis showed that, in addition to the strong association with HAM/TSP, mortality was directly related to age, likely representing longer disease duration, and to the number of hospitalizations. Unexpectedly, no significant association was observed between mortality and HIV co‐infection, which is potentially attributable to the implementation of HAART [31]. However, the small sample size of HIV/HTLV‐1 co‐infected patients (*n* = 37) limited definitive conclusions, and larger studies are needed to elucidate this issue.

The sex distribution was consistent with previous reports, with predominance of females [32], and the relatively low median EDSS score and baseline HTLV‐1 PVL reflected the combined analysis of individuals with HAM/TSP and asymptomatic carriers. As expected, no association was identified between sex and mortality. Notably, HTLV‐1 PVL was a significant predictor for mortality in the overall cohort or among symptomatic individuals. High HTLV‐1 PVL has been shown to increase the inflammatory burden and play a role in the development of HAM/TSP [10, 33]. This corroborates a prior study conducted in Guinea–Bissau, showing that higher HTLV‐1 PVL predicted death [34].

This study was conducted in one of the largest cohorts of adults living with HTLV‐1, classified consistently as symptomatic or asymptomatic. Nonetheless, potential distortions in hospitalization rates due to heterogeneous hospital access over three decades cannot be excluded. The open and unstructured cohort design also limited the identification of additional factors influencing morbidity and mortality in HTLV‐1 infection.

In conclusion, UTIs, pneumonia, and skin infections emerged as major contributors to morbidity and mortality among individuals living with HTLV‐1, particularly in patients with HAM/TSP. This highlights the significance of implementing preventive strategies, including intermittent catheterization protocols and interventions aimed at reducing the risk of pressure ulcers. In addition, increased susceptibility to respiratory infections in this group also highlights the need to consider HTLV‐1 infection when recommending influenza and pneumococcal vaccination.

## Author Contributions

Conceptualization: Elizabeth Souza Neves, Ana Claudia Celestino Bezerra Leite, and Abelardo Queiroz Campos Araújo. Methodology: Otavio Melo Espíndola. Investigation: Ana Claudia Celestino Bezerra Leite, Marcus Tulius Teixeira Silva, Marco Antonio Sales Dantas Lima, Abelardo Queiroz Campos Araújo, Rafael Osellame, Pedro Abrahão Pinheiro Guimarães, Ricardo Vezzani Batista, and André Luiz Anjos Oliveira. Data curation: Elizabeth Souza Neves and Ana Claudia Celestino Bezerra Leite. Formal analysis: Raquel de Vasconcelos Carvalhaes de Oliveira. Visualization: Otavio Melo Espíndola and Raquel de Vasconcelos Carvalhaes de Oliveira. Writing (Original Draft): Elizabeth Souza Neves and Raquel de Vasconcelos Carvalhaes de Oliveira. Writing (Review and Editing): Elizabeth Souza Neves and Otavio Melo Espíndola. All authors have read and agreed to the published version of the manuscript.

## Conflicts of Interest

The authors declare no conflicts of interest.

## Data Availability

The data that support the findings of this study are available from the corresponding author upon reasonable request.
